# Anti-thyroid peroxidase antibody in stroke localization: exordium doorway of preliminary findings in thyroidology?

**DOI:** 10.1590/1806-9282.20240447

**Published:** 2024-09-02

**Authors:** Nurce Cilesizoglu Yavuz, Demet Seker, Demet Sengul, Ilker Sengul, Esma Cinar, José Maria Soares

**Affiliations:** 1 Giresun University, Faculty of Medicine, Department of Physical Medicine and Rehabilitation – Giresun, Turkey.; 2 Giresun University, Faculty of Medicine, Department of Neurology – Giresun, Turkey.; 3 Giresun University, Faculty of Medicine, Department of Pathology – Giresun, Turkey.; 4 Giresun University, Faculty of Medicine, Division of Endocrine Surgery – Giresun, Turkey.; 5 Giresun University, Faculty of Medicine, Department of General Surgery – Giresun, Turkey.; 6 Universidade de São Paulo, Hospital das Clínicas, Faculdade de Medicina, Departamento de Obstetrícia e Ginecologia, Disciplina de Ginecologia, Laboratório de Ginecologia Estrutural e Molecular – São Paulo (SP), Brazil.

**Keywords:** Stroke, Thyroid gland, Peroxidase, Thyroidologists, Pathology

## Abstract

**OBJECTIVE:**

Stroke is a chronic health problem that affects all areas of life. The presence of thyroid autoantibodies can augment the severity of stroke. The aim of this work is to investigate whether there is a relationship between the site of stroke involvement and the anti-thyroid peroxidase antibody (anti-TPO) or not. This is the first study in the English-language literature.

**METHODS:**

A total of 39 patients with a diagnosis of acute ischemic stroke were included, and the cases under 18 years of age with an infection and the ones with autoimmune diseases other than Hashimoto's thyroiditis were excluded from the study design. The patients’ age, gender, smoking status, comorbid conditions, and stroke localization in brain imaging were recorded. The region involving the anterior circulation area originating from the internal carotid artery was evaluated as anterior, and the region possessing the vertebrobasilar circulation area from the vertebral arteries was considered posterior involvement. Thyroid-stimulating hormone (TSH), triiodothyronine (T3), thyroxine (T4), triglyceride, high-density lipoprotein (HDL), low-density lipoprotein (LDL), C-reactive protein (CRP), sedimentation, and anti-TPO were retrospectively analyzed.

**RESULTS:**

As a consequence, gender distribution, smoking, comorbid conditions, TSH, T3, T4, triglyceride, HDL, LDL, CRP, and sedimentation did not differ significantly, while the age of the posterior-located stroke was lower than that of the cases with the anterior. The anti-TPO value was significantly lower in posterior-located strokes than in the anterior system.

**CONCLUSION:**

In summary, the anti-TPO value was recognized as higher in the anterior stroke localization. Thyroiditis and accompanying anti-TPO autoantibody positivity are conditions that should not be ignored by thyroidologists and thyroid-health providers.

## INTRODUCTION

Hashimoto's thyroiditis (HThy) is an autoimmune thyroid disease and the most common cause of hypothyroidism in developed countries. Both cellular and humoral responses play a role in pathogenesis. Initially, activation of thyroid-specific CD4+ T cells stimulates the generation of CD8+ cytotoxic T cells and autoantibodies. Cytotoxic T cells are mainly responsible for parenchymal destruction, and sensitized B cells secrete antibodies that block the action of TSH, contributing to the development of hypothyroidism. As a result of the presentation of thyroid antigens released by tissue destruction to the immune system, antithyroglobulin (anti-Tg) and antithyroid peroxidase antibodies (anti-TPO) are released into the circulation, which are helpful in diagnosis. HThy is associated with many systemic diseases as well as local effects, which can be essential and affect the person's vital functions. HThy is blamed for many significant cardiovascular^1^ and cerebrovascular diseases^2^.

Stroke has a vital place in cerebrovascular diseases and is a chronic health problem that affects all areas of life. It is the world's most common cause of death after heart disease and cancer, and the third most common cause of death. A stroke is defined as a sudden and rapidly developing loss of motor control, sensation deficit, balance disorder, speech, and cognition from dysfunction to coma for more than 24 h^3^. Autoimmunity, particularly anti-TPO levels in the thyroid, has been raised as associated with the development of intracranial stenosis even in euthyroid patients^4^. The presence of thyroid autoantibodies without hypothyroidism has been reported as a condition that augments the severity of stroke^5^. The relationship between one of these antibodies, anti-TPO, and stroke localization has not been clearly demonstrated.

Evaluations regarding anterior and posterior strokes and clinical, etiological, radiological, and outcome factors are scarce. Approximately 80% of cerebral blood flow is obtained from the anterior circulation, and 20% is from the posterior. Arterial anatomy and location of occlusion show significant differences in anterior and posterior strokes^6^. Data on stroke mechanisms in posterior and anterior strokes are conflicting. There are studies supporting the fact that embolism is more common in the posterior and lacunae in the anterior^7,8^. To the best of our knowledge, the present study is the first work in the English-language literature to evaluate the liaison between anti-TPO levels and the site of stroke involvement.

In this study, in which we investigated the relationship of anti-TPO with strokes in the anterior and posterior circulation regions of the brain, we found that anti-TPO levels were higher in strokes in the anterior circulation systems.

## METHODS

### Study design and participants

A total of 39 patients with a diagnosis of acute ischemic stroke were included in this study. The inclusion criteria were possessed a diagnosis of acute ischemic stroke, being under 18 years of age, and having an infection. The exclusion criteria were having autoimmune diseases such as systemic lupus erythematosus or rheumatoid arthritis and having positive autoantibodies other than thyroid autoantibodies, which may be a marker of autoimmunity. All participants provided written informed consent, and the study was performed in accordance with the Declaration of Helsinki.

The patients’ age, gender, smoking status, comorbid conditions, and stroke localization in brain imaging were recorded. In the laboratory, thyroid stimulating hormone (TSH), triiodothyronine (T3), thyroxine (T4), anti-TPO, and other laboratory parameters that constitute risk factors for stroke, triglyceride, high-density lipoprotein (HDL), low-density lipoprotein (LDL), C-reactive protein (CRP), and sedimentation levels were measured. Participants’ thyroid function test results were consistent with euthyroidism. Diffusion magnetic resonance imaging (MRI) was used as the brain imaging method. Anterior involvement, which originates from the internal carotid artery, and posterior involvement, which is involved from the vertebrobasilar circulation, were evaluated with an MRI, which was performed at the time of diagnosis.

### Sample size and statistical analyses

G*Power (V3.1) software (Informer Technologies, Inc., Los Angeles, CA, USA) was used to calculate the required sample size. To the best of our knowledge, there is no study comparing the anterior and posterior regions in the English-language literature. We set the effect size to 0.95. Based on a power of 80% and a 5% level of significance, the total sample size required was calculated as 38. In the descriptive statistics of the data, mean, standard deviation, median, minimum, maximum, frequency, and ratio values were used. The distribution of variables was measured with the Kolmogorov-Smirnov test. An independent sample t-test and a Mann-Whitney U test were used to analyze the quantitative independent data. Afterward, the chi-square test was utilized in the analysis of qualitative independent data, and the Fischer test was performed when the chi-square test conditions were not met. The SPSS 28.0 program was used in the analysis. The receiver operating characteristic (ROC) curve was created by selecting options for the anti-TPO threshold value depending on different sensitivity-specificity characters. The choice of optimum cut-off sensitivity and specificity was made considering the case where the Youden index is the largest.

## RESULTS

Gender distribution, smoking, comorbid conditions, and laboratory values such as TSH, T3, T4, triglyceride, HDL, LDL, CRP, and sedimentation did not differ significantly in the group with stroke localization in the anterior and posterior systems. The age of the posterior-located stroke patients was lower than that of the cases with the anterior system. The anti-TPO value was significantly lower in the group with stroke localization in the posterior system than in the group with stroke localization in the anterior system (Table 1).

**Table 1:** Demographic, clinical, and laboratory characteristics according to the stroke involvement site.

		Stroke localization								p	
		Anterior (n=29)				Posterior (n=10)					
		Mean±SD/n-%			Median	Mean±SD/n-%			Median		
Age		77.3	±	10.4	80.0	68.5	±	13.6	69.5	0.039	t
Gender	Female	15		51.7%		7		70.0%		0.315	X^ 2^
	Male	14		48.3%		3		30.0%			
Smoking	No	18		62.1%		7		70.0%		0.884	X^ 2^
	Ex-smoker	3		10.3%		0		0.0%			
	Yes	8		27.6%		3		30.0%			

Diabetes	(-)	18		62.1%		8		80.0%		0.300	X^ 2^
	(+)	11		37.9%		2		20.0%			
Hypertension	(-)	4		13.8%		4		40.0%		0.077	X^ 2^
	(+)	25		86.2%		6		60.0%			
Cardiovascular disease	(-)	16		55.2%		5		50.0%		0.777	X^ 2^
	(+)	13		44.8%		5		50.0%			
TSH		1.9	±	1.6	1.3	2.4	±	2.1	1.9	0.520	^m^
T3		2.6	±	0.5	2.6	2.3	±	0.4	2.4	0.209	t
T4		1.2	±	0.2	1.2	1.1	±	0.2	1.1	0.082	t
Anti-TPO		21.3	±	28.7	12.4	11.3	±	7.9	9.0	0.037	m
Triglyceride		127.7	±	55.9	124.0	162.6	±	63.9	137.0	0.109	t
HDL (mg/dL)		41.7	±	9.1	43.0	43.1	±	12.2	45.5	0.702	t
LDL (mg/dL)		104.1	±	32.8	105.0	111.3	±	43.1	120.0	0.584	t
CRP (mg/L)		23.1	±	30.0	12.7	52.4	±	128.5	4.6	0.139	m
Sedimentation		36.8	±	17.5	35.0	28.0	±	23.8	19.5	0.062	m

t Independent sample t-test

m Mann-Whitney U test

X^ 2^ Ki-kare test (Fischer's exact test).

**Figure 1:**
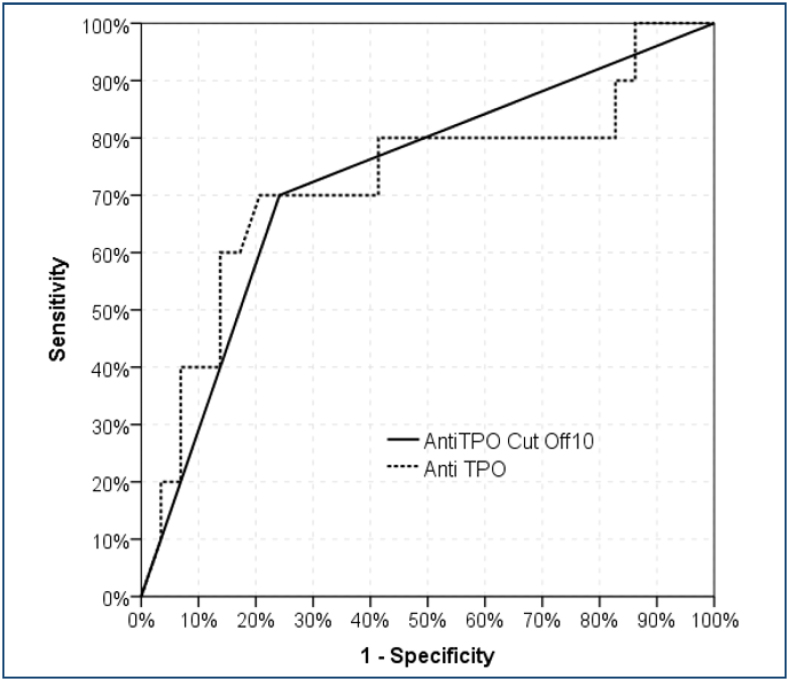
The ROC curves of the anti-TPO w/wo 10 cut off value to differentiate the patients with stroke in the anterior and posterior systems.

A significant [area under the curve: 0.724 (0.518–0.930)] effectiveness of the anti-TPO value was observed in the differentiation of patients with stroke localization from the anterior and posterior systems. A significant [area under the curve: 0.729 (0.540–0.919)] efficacy of the anti-TPO 10 cut-off value was observed in the differentiation of patients with stroke localization from the anterior and posterior systems (Figure 1). At the anti-TPO 10 cut-off value, the sensitivity was 70.0%, the positive prediction was 50.0%, the specificity was 75.9%, and the negative prediction was 88.0% in separating patients with stroke localization from the anterior and posterior systems (Table 2).

**Table 2:** The receiver operating characteristic analysis in terms of anti-thyroid peroxidase antibody cut-off 10.

		Area under the curve		% 95 Confidence Interval			p
Anti-TPO		0.724		0.518	-	0.930	0.037
Anti-TPO cut-off 10		0.729		0.540	-	0.919	0.032
		Anterior	Posterior				%
Anti-TPO	<10	22	3	Sensitivity			70.0%
	>10	7	7	Positive prediction rate			50.0%
				Specificity			75.9%
				Negative prediction rate			88.0%

## DISCUSSION

Hashimoto's thyroiditis is considered an autoimmune thyroid disease characterized by high antibody titers, which frequently affects females and is most often diagnosed between the ages of 30 and 50. HThy may be accompanied by many clinical findings, both locally and systemically. However, studies have shown that various systemic problems are more common in the presence of thyroid autoantibodies in patients without hypothyroidism. There are various studies in the literature that have shed light on the recent relationship between increased stroke cases and thyroid autoantibody positivity. There is a relationship between young stroke cases and thyroid autoantibody-positive euthyroid patients^9^. It has been shown that anti-TPO plays a role in arterial remodeling in patients with intracranial stenosis, which is the most common cause of ischemic stroke worldwide^4^. This study exhibits that anti-TPO levels are associated with stroke with anterior circulation involvement, independent of thyroid functions.

Thyroid hormones are modulators that critically affect different aspects of tissue development. The brain is a vital target tissue for thyroid hormones, and deficiency or excess in thyroid hormone levels disrupts neuronal organization during embryonic and adult life, and cognitive functionality may be lost. Research results explaining the relationship between acute cerebral and cardiac diseases and thyroid hormones have been increasing recently. Ischemic stroke is a significant neurological disease and a major cause of disability and death. Neurological findings vary according to the size and location of the infarction. Temporary or permanent occlusion of cerebral vessels causes significant functional losses in patients after neuronal damage^10-12^.

Atherosclerosis is always accompanied by an autoimmune response, which has a secondary autoimmune component, and a self-antigen-specific adaptive immune response plays a role in the disease. The improvement of intracranial stenosis associated with Graves’ disease after high-dose methylprednisolone and plasmapheresis treatment and the stabilization of Moya Moya disease with hyperthyroidism after plasmapheresis have been demonstrated in case reports^13,14^. Elevation of anti-TPO, a substantial thyroid autoantibody in thyroidology^15-19^, was found in patients with euthyroid young intracranial stenosis in a study^9^. Inappropriate autoimmune responses trigger vascular damage, contributing to endothelial dysfunction and atherosclerosis^20^. Xiang et al.^21^ advocated that endothelium-dependent arterial dilatation is impaired in patients with euthyroid autoimmune thyroiditis. Piga et al.^22^ stated that brain perfusion was reduced in patients with autoimmune thyroiditis. Compared to healthy donors, it has been shown that higher rates of interferon-γ (IFN-γ) are produced from T cells of patients with high anti-TPO titers by Laurat and colleagues^23^. It has been emphasized in studies that the presence of aberrant T cells in atherosclerosis lesions and inappropriate IFN-γ release from these T cells play a momentous role in the development of atherosclerosis^24^, which results point to a link between thyroid autoantibodies and atherosclerosis. Tanaka et al.^25^ revealed that thyroid antibodies were associated with stenotic lesions in the terminal portion of the internal carotid artery. In this respect, in our study showing that anti-TPO positivity is at high titers in patients with stroke with anterior system involvement, we think that interventions to reduce autoimmunity against the thyroid gland may also prevent the recurrence of a new stroke that may develop. However, these are preliminary findings, and comprehensive studies are required in the future.

### Limitations

Our study has some limitations. The small numeric values of patients, the fact that the patient diagnosed with stroke had brain imaging at the time of diagnosis, and the lack of serial measurements of anti-TPO values with previous imaging methods can be considered limitations. Non-HDL cholesterol components, which are an important cardiovascular risk factor, were not included in the laboratory parameters, and the relationship between anti-TPO values and clinical findings was not evaluated, which is an additional limitation. Furthermore, there may be anatomical variations between people that were not taken into account in the study.

## CONCLUSION

In summary, the prevention of stroke cases, which significantly affects public health, is an issue that should be considered. In this respect, studies supporting the relationship between autoinflammation and atherosclerosis, which is the leading cause of stroke, are essential. The findings of this study point out that the anti-TPO value was significantly higher in the anterior stroke localization. Finally, thyroiditis and accompanying anti-TPO autoantibody positivity are conditions that should not be ignored by thyroidologists and thyroid-health providers.
